# Diversity of dinoflagellate assemblages in coastal temperate and offshore tropical waters of Australia

**DOI:** 10.1186/s12862-021-01745-5

**Published:** 2021-02-15

**Authors:** Tahnee Manning, Arjun Venkatesh Thilagaraj, Dmitri Mouradov, Richard Piola, Clare Grandison, Matthew Gordon, Jeff Shimeta, Aidyn Mouradov

**Affiliations:** 1 School of Science, RMIT University, Melbourne, VIC Australia; 2 Personalised Oncology Division, The Walter and Eliza Hall Institute of Medical Research, Parkville, VIC Australia; 3 Maritime Division, Defence Science & Technology Group, Fishermans Bend, Canberra, VIC, Australia

**Keywords:** Bioluminescence, Dinoflagellates, eDNA, Genotyping, Harmful algal bloom, High throughput sequencing

## Abstract

**Background:**

Dinoflagellates are a ubiquitous and ecologically important component of marine phytoplankton communities, with particularly notable species including those associated with harmful algal blooms (HABs) and those that bioluminesce. High-throughput sequencing offers a novel approach compared to traditional microscopy for determining species assemblages and distributions of dinoflagellates, which are poorly known especially in Australian waters.

**Results:**

We assessed the composition of dinoflagellate assemblages in two Australian locations: coastal temperate Port Phillip Bay and offshore tropical waters of Davies Reef (Great Barrier Reef). These locations differ in certain environmental parameters reflecting latitude as well as possible anthropogenic influences. Molecular taxonomic assessment revealed more species than traditional microscopy, and it showed statistically significant differences in dinoflagellate assemblages between locations. Bioluminescent species and known associates of HABs were present at both sites. Dinoflagellates in both areas were mainly represented by the order Gymnodiniales (66%—82% of total sequence reads). In the warm waters of Davies Reef, Gymnodiniales were equally represented by the two superclades, Gymnodiniales sensu stricto (33%) and Gyrodinium (34%). In contrast, in cooler waters of Port Phillip Bay, Gymnodiniales was mainly represented by Gyrodinium (82%). In both locations, bioluminescent dinoflagellates represented up to 0.24% of the total sequence reads, with *Protoperidinium* the most abundant genus. HAB-related species, mainly represented by *Gyrodinium*, were more abundant in Port Phillip Bay (up to 47%) than at Davies Reef (28%), potentially reflecting anthropogenic influence from highly populated and industrial areas surrounding the bay. The entire assemblage of dinoflagellates, as well as the subsets of HAB and bioluminescent species, were strongly correlated with water quality parameters (R^2^ = 0.56–0.92). Significant predictors differed between the subsets: HAB assemblages were explained by salinity, temperature, dissolved oxygen, and total dissolved solids; whereas, bioluminescent assemblages were explained only by salinity and dissolved oxygen, and had greater variability.

**Conclusion:**

High-throughput sequencing and genotyping revealed greater diversity of dinoflagellate assemblages than previously known in both subtropical and temperate Australian waters. Significant correlations of assemblage structure with environmental variables suggest the potential for explaining the distribution and composition of both HAB species and bioluminescent species.

## Background

Dinoflagellates are single-celled protists ubiquitously found in freshwater and marine environments where they occupy many ecological niches. They play significant roles from primary producers through to detritus feeders. Dinoflagellates belong to the class Dinophyceae, which consists of 117 genera including about 4000 free-living species, plus 500 species with parasitic or mutualist symbiotic lifestyles representing essential symbionts of reef-building corals [1]. Living under diverse environmental conditions, dinoflagellates have developed high complexity in behaviour, nutritional modes (photoautotrophic, heterotrophic, and mixotrophic) and broad morphological diversity, ranging from 5 µm to 2 mm.

Dinoflagellate species are known to be a major component of harmful algal blooms (HABs) with a set of physical and chemical effects causing a significant hazard to ecosystems, fisheries, and animal and human health [2]. Physical effects can lead to depletion of oxygen in the water and an increase in viscosity due to the excretion of mucilage. Chemical consequences include the secretion of toxins, that can trigger cell necrosis, affect cell wall penetrability, and suppress the digestive system of other marine organisms as well as potentially humans via either direct exposure or consumption, for example, affected shellfish [3]. HABs are naturally occurring global phenomena but can be triggered by anthropogenic activities, including shipping, eutrophication, and global warming.

There are HAB forming species that also exhibit bioluminescence such as *Noctiluca scintillans*, although not all bioluminescent dinoflagellates are considered HABs. Sixty-eight dinoflagellate species have evolved bioluminescence, the function of which is still not entirely clear [4]. Bioluminescence is a result of the biochemical process of interaction between a luciferase protein and its substrate, a luciferin, in the presence of a luciferin-binding protein [5]. One of the most widely accepted hypotheses proposes that luminescence increases the survival of dinoflagellates, acting as a ‘‘burglar alarm’’ [6]. The role of bioluminescence as a defence against zooplankton grazers was shown by increased bioluminescence of *Lingulodinium polyedra* in response to amides produced by their copepod grazers [7]. As a result of bioluminescence, dinoflagellates can successfully persist within marine phytoplankton communities dominated by the similarly sized competitors, diatoms and green algae. However, the existence of non-bioluminescent *N. scintillans* blooms in the northeast Pacific coast of USA, associated with mutation of the luciferase (*lcf)* gene, disarms this species against their predators [8]. The ecological significance of this phenomenon and a long term existence or disappearing of this mutant will give more information about the importance of bioluminescence as a defence mechanism against predators. Changes in intensity of bioluminescence can be used as a sensitive indicator for the distribution of planktonic biomass in response to physical and chemical changes in surface waters [9], and may offer the possibility of predicting HAB events in known bloom forming localities [10].

In recent years, omics technologies, such as genomics, transcriptomics, proteomics, and metabolomics, have been extensively applied to study marine dinoflagellates, uncovering essential molecular pathways, such as in toxin biosynthesis, symbiosis, lipid biosynthesis, and HAB formations [11, 12]. Application of molecular techniques to the taxonomy of dinoflagellates such as high-throughput sequencing (HTS) genotyping has led not only to advanced methods for identification of these species but also provides a new tool for understanding their evolutionary complexity and diversity as well as their roles in marine ecological networks [13–18].

Regions targeted for assessment of dinoflagellate diversity include *lcf* [19], the nuclear ribosomal internal transcribed spacer region (*ITS2*) [15, 17, 20], cytochrome *c* oxidase 1 (*COI*) [21], heat shock protein (HSP90) [22], 28S rRNA and 18S rRNA [13, 23–30]. 18S rRNA and 28S rRNA have proven highly popular for assessing the diversity of many marine microorganisms [13, 24–26, 28–31]. Recently, the DinoREF dinoflagellate database, has been developed based on V4 regions of 18S rRNA sequences [32]. Originally, this database was used to assess the diversity and seasonal changes of dinoflagellates in the Gulf of Naples [27].

The east Australian coastline ranges from the tropical waters of the Great Barrier Reef and Coral Sea to the temperate waters of the Tasman Sea, with water movement driven north to south via the East Australian Current. Because of climate change, many marine species are shifting to higher latitudes [12, 33]. This has led to changes in the diversity of marine populations in these locations, and has triggered extensive study linking changes in planktonic communities to changes in physical, biological and chemical oceanographic conditions [3, 13–17, 34–37].

**Fig. 1:**
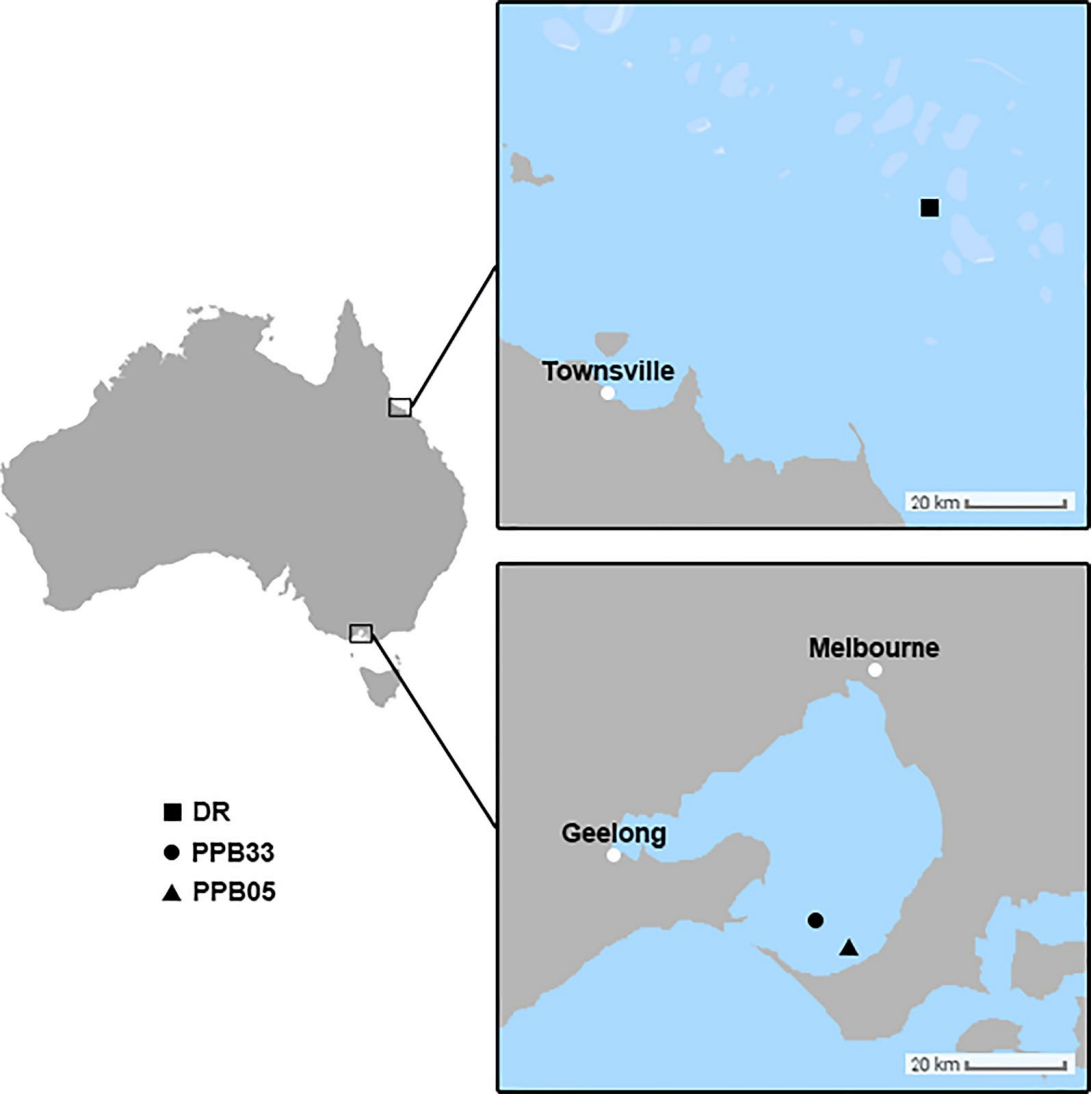
Locations of study sites, Davies Reef (DR), Queensland, and Port Phillip Bay (PPB), Victoria, Australia. Maps created using Adobe Photoshop, version 21.2.0 (https://www.adobe.com/au/products/photoshop.html)

Davies Reef (DR) is part of the Great Barrier Reef system within the sub-tropic zone in the Coral Sea, approximately 70 km from shore [38, 39] (Fig. 1). In contrast, Port Phillip Bay (PPB) is located 2000 km south within the temperate zone, bordered by Melbourne, the capital city of Victoria, that accommodates 4.96 million people (Fig. 1). There are busy ports, with over 2500 commercial vessels visiting port of Melbourne in 2018–2019 [40] as well as industrial areas contained within the region. As a result, PPB is subject to both direct and indirect anthropogenic influence such as nutrient runoff and industrial pollution [41] that can lead to the formation of HABs. There is no significant upwelling in the region.

In this study, we assessed the diversity and composition of dinoflagellate assemblages in two different Australian ecosystems: offshore sub-tropical waters at DR and the southern coastal temperate PPB. Taxonomic identification of dinoflagellate diversity was assessed using conventional light microscopy as well as by application of HTS of 18S rRNA amplicons of DNA extracted from environmental samples (eDNA). Correlations between dinoflagellate assemblage structure and environmental factors were found.

## Results

### Taxonomic identification of dinoflagellates

Samples were collected from two different Australian locations: offshore tropical waters of Davies Reef, and temperate coastal waters located close to Melbourne city in Port Phillip Bay, Victoria, during June of 2018 (Austral winter) (Fig. 1). Conventional, microscopy based taxonomic identification of the main planktonic representatives (limited to diatoms and dinoflagellates) identified from samples collected at the PPB and DR sites are shown in Additional file 1: Tables S1 and S2. Other taxonomic groups, such as Chrysophytes, Prymnesiophytes, Cryptophytes, Prasinophytes, Euglenophyta, and Cyanoprokaryota, were represented in much smaller numbers and are not shown in these tables. Dinoflagellates collected in offshore tropical waters of DR (water temperature 24 °C) were mainly represented by *Gyrodinium* spp. (up to 1.0 × 10^3^ cells ml^−1^)*, Gymnodinium* spp. *(*up to 6.0 × 10^3^ cells ml^−1^), and *Heterocapsa* spp. (up to 1.0 × 10^4^ cells ml^−1^), which were found in all analysed samples. The same species were also dominant in cooler, coastal temperate waters of PPB (water temperature 14 °C). Luminescent dinoflagellates in PPB were mainly represented by *Noctiluca scintillans* (up to 1.5 × 10^2^ cells ml^−1^), with bioluminescence visible at night at the collection areas.

**Fig. 2:**
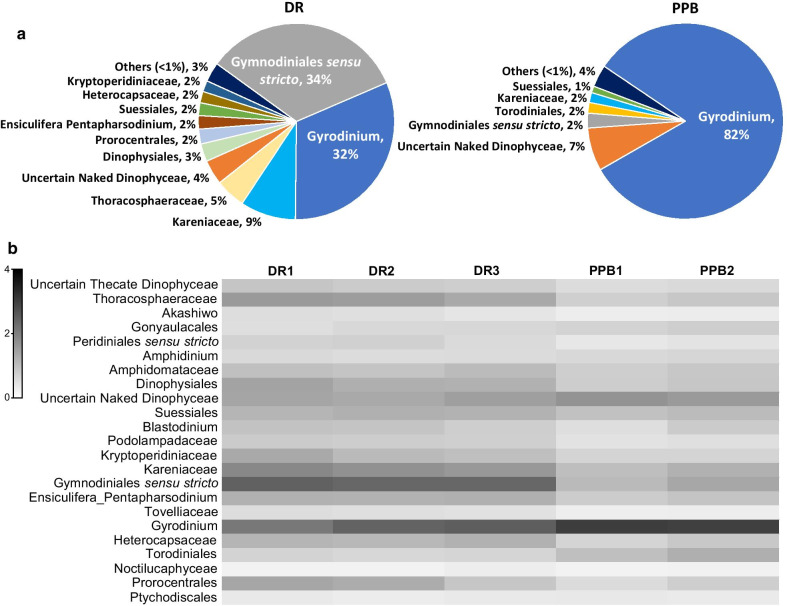
Dinoflagellate superclades identified at Davies Reef (DR) and Port Phillip Bay (PPB). **a** Superclade relative abundances of sequence reads ≥ 1% for DR and PPB. **b** Relative abundance of sequence reads, fourth-root transformed, of superclades for each sample at each location for all 23 superclades. The scale represents the shading intensity within the matrix, indicating the fourth-root transformed relative abundance of sequence reads for superclades

**Fig. 3:**
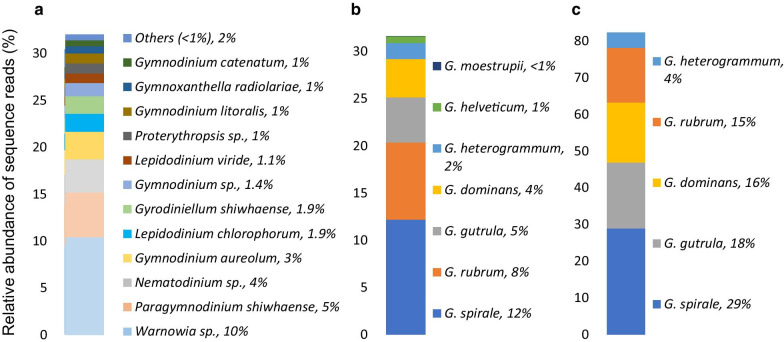
Relative abundance of species belonging to superclades Gymnodiniales sensu stricto and Gyrodinium. Davies Reef (**a** and **b**) and Port Phillip Bay (**c**). A threshold of ≥ 1% relative abundance of sequence reads was applied. Note that the scale of y-axis has been adjusted in facet C

For molecular taxonomy, 18S rRNA amplicons (63,619–389,041 per sample) of eDNA collected in DR and PPB were matched against the DinoREF database, revealing 4,331 ribotypes based on 98% sequence similarity. The identified dinoflagellates across both DR (409 species and a further 45 dentified to genus only) and PPB (404 species and a further 44 identified to genus only) were represented by 8 orders, 23 superclades, 40 families and 150 genera, based on taxonomy reviewed in [32] (Additional file 1: Tables S3, S4, and S5). For the graphical representation of the most abundant superclades, genera, and individual species identified in both locations, an abundance value of ≥ 1% of sequence reads was used as a threshold. DR samples contained representatives of eleven superclades, composing 97% of the total sequence reads (Fig. 2a). Superclade Gymnodiniales sensu stricto (one of the largest dinoflagellate clades containing 16 genera) and Gyrodinium (superclade represented by a single genus, *Gyrodinium*) comprised 34% and 32% of sequence reads, respectively. Nine other superclades showed ≥ 1% of sequence reads (Additional file 1: Tables S4 and S5). The same threshold applied to PPB samples showed that 96% of the total assemblage consisted of just six superclades, with Gyrodinium comprising 82% of the total sequence reads. Gymnodiniales sensu stricto represented just 2% of the sequence reads. The shade plot (Fig. 2b) displays all twenty-three superclades' relative abundances, fourth-root transformed to capture both highly represented and rarely represented species, for samples at each location. The overall profiles across samples for PPB indicated the dominance of the genus *Gyrodinium*. Similarly, for DR samples, the dominance of two superclades, Gymnodiniales sensu stricto and Gyrodinium, can be observed across all samples (Fig. 2b). The graphic representation of these two superclades identified in both locations (with an abundance value of ≥ 1% of sequence reads) is shown in Fig. 3 and Additional file 1: Table S5. In DR, Gymnodiniales sensu stricto was represented by forty-four species, from which just twelve species showed abundance ≥ 1% of sequence reads. The genus *Gyrodinium* was represented by seven species, from which six showed abundance ≥ 1%: *G. spirale* (12%), *G. rubrum* (8%), *G. gutrula* (5%), *G. dominans* (4%), *G. heterogrammum (2%)* and *G. helveticum* (1%). In PPB, *Gyrodinium* was comprised of five species with abundance ≥ 1%: *G. spirale* (29%), *G. gutrula* (18%), *G. dominans* (16%), *G. rubrum* (15%) and *G. heterogrammum* (4%).

**Fig. 4:**
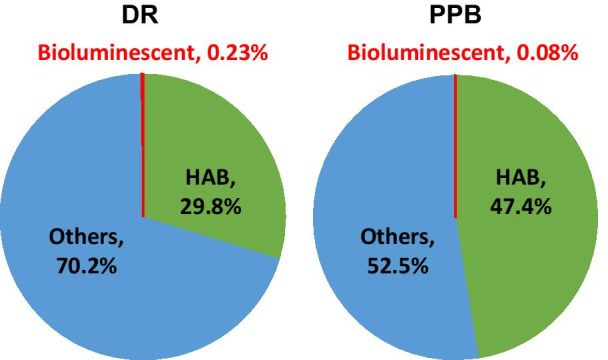
Dinoflagellate species identified at DR and PPB. Values represent mean relative abundance of sequence reads (%)

Identified species were categorised as belonging to the HAB-related and/or bioluminescent species groups (Additional file 1: Table S6). Only a small percentage of identified species were bioluminescent (0.23% and 0.08% for DR and PPB, respectively), but a larger number were HAB-related (29.8% and 47.4% of total assemblages for DR and PPB, respectively) (Fig. 4). Eleven of the HAB-related species belong to the family of bioluminescent dinoflagellates, including five *Alexandrium* spp., two *Gonyaulax* and *Pyrodinium* spp. as well as *Lingulodinium polyedra* and *Noctiluca scintillans* (Additional file 1; Table S6).

### Bioluminescent dinoflagellates

A list of bioluminescent dinoflagellate species and the relative abundances of sequence reads for each location is shown in Table 1. In both locations, *Protoperidinium* was the most abundant genus, being represented by 11 species, with *P. pellucidum*, the most abundant in DR (0.04%) and *P. pallidum* in PPB (0.01%). *Alexandrium, Tripos and Pyrocystis* were represented by 5, 3 and 3 species, respectively, in both locations. *Gonyaulax, Polykrikos* and *Pyrodinium* were represented by 2 species. *Polykrikos kofoidii* was the most representative bioluminescent dinoflagellate species identified in both locations, with concentrations over threefold higher in DR (0.10%) than in PPB (0.03%).

**Table 1:** Relative abundance of sequence reads (%) of bioluminescent dinoflagellate species

DR	%	PPB	%
*Polykrikos kofoidii*	0.10241	*Polykrikos kofoidii*	0.03014
*Protoperidinium pellucidum*	0.04454	*Protoperidinium pallidum*	0.01365
*Polykrikos schwartzii*	0.02687	*Alexandrium fundyense Group I*	0.00733
*Protoperidinium pallidum*	0.01386	*Pyrodinium bahamense var. compressum*	0.00689
*Alexandrium fundyense Group I*	0.01271	*Protoperidinium punctulatum*	0.00313
*Pyrodinium bahamense var. compressum*	0.00410	*Alexandrium affine*	0.00290
*Gonyaulax spinifera*	0.00365	*Noctiluca scintillans*	0.00253
*Noctiluca scintillans*	0.00307	*Fragilidium sp.*	0.00219
*Protoperidinium punctulatum*	0.00299	*Protoperidinium conicum*	0.00188
*Protoperidinium conicum*	0.00227	*Polykrikos schwartzii*	0.00154
*Alexandrium affine*	0.00197	*Tripos furca*	0.00126
*Fragilidium sp.*	0.00135	*Lingulodinium polyedra*	0.00094
*Pyrodinium bahamense var. bahamense*	0.00125	*Pyrocystis lunula*	0.00089
*Alexandrium ostenfeldii*	0.00118	*Ceratocorys horrida*	0.00088
*Pyrocystis lunula*	0.00116	*Alexandrium monilatum*	0.00085
*Lingulodinium polyedra*	0.00108	*Alexandrium ostenfeldii*	0.00084
*Tripos furca*	0.00103	*Alexandrium tamarense Group III*	0.00071
*Ceratocorys horrida*	0.00087	*Pyrodinium bahamense var. bahamense*	0.00069
*Protoperidinium excentricum*	0.00087	*Protoperidinium excentricum*	0.00057
*Alexandrium tamarense Group III*	0.00080	*Protoperidinium pellucidum*	0.00051
*Alexandrium monilatum*	0.00076	*Gonyaulax spinifera*	0.00043
*Protoperidinium divergens*	0.00055	*Protoperidinium divergens*	0.00031
*Gonyaulax polygramma*	0.00055	*Protoperidinium leonis*	0.00021
*Pyrocystis sp.*	0.00029	*Gonyaulax polygramma*	0.00020
*Protoperidinium leonis*	0.00028	*Protoperidinium crassipes*	0.00009
*Pyrocystis noctiluca*	0.00026	*Protoperidinium claudicans*	0.00008
*Tripos fusus*	0.00025	*Pyrocystis sp.*	0.00007
*Pyrophacus steinii*	0.00017	*Pyrophacus steinii*	0.00006
*Protoperidinium crassipes*	0.00014	*Protoperidinium depressum*	0.00006
*Protoperidinium claudicans*	0.00010	*Tripos horridus*	0.00005
*Protoperidinium depressum*	0.00010	*Protoperidinium pentagonum*	0.00004
*Protoperidinium pentagonum*	0.00009	*Tripos fusus*	0.00004
*Tripos horridus*	0.00001	*Pyrocystis noctiluca*	0.00003
Total:	0.23156		0.08197

*DR* Davies Reef, *PPB* Port Phillip Bay

### HAB-related dinoflagellates

**Fig. 5:**
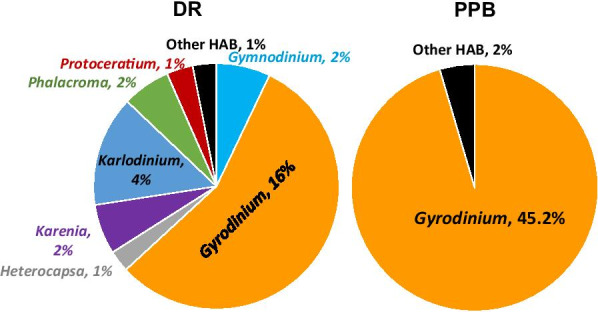
HAB-related genera and species at Davies Reef (**a**) and Port Phillip Bay (**b**). A threshold of ≥ 1% relative abundance of sequence reads was applied

HAB-related dinoflagellates identified in both locations were represented by 26 genera, which included 92 and 90 species comprising 28.2% and 47.2% of all sequence reads, for DR and PPB, respectively (Additional file 1: Tables S6 and S7). HAB-related genera with an abundance ≥ 1% of sequence reads are shown in Fig. 5. At DR, *Gyrodinium* members were among the most abundant (16%), with a significantly lower representation of other genera, such as *Karlodinium* (4.2%), *Gymnodinium* (2.05%), *Karenia* (1.9%), and *Phalacroma* (2.0%). *Heterocapsa, Protoceratium,* and the total number of the remaining HAB representatives counted as 1%. These genera were represented by different numbers of species, with the most abundant, *Gyrodinium,* including *Gyrodinium spirale* (12.1%) and *Gyrodinium dominans* (4.0%) (Additional file 1: Tables S6 and S7)*.* However, the least abundant was *Prorocentrum*, containing 11 species. *Alexandrium*, a common and abundant member of most HAB communities, included 17 species in the DR location (only 0.05%). The HAB-related dinoflagellate species in PPB were mainly represented by two *Gyrodinium* species: *Gyrodinium spirale* (29%) and *Gyrodinium dominans* (16%). The contribution of other HAB-related genera, represented by 88 species, was just 2% of sequence reads (Fig. 4, Additional file 1: Tables S6 and S7).

### Assemblage patterns and correlations with environmental variables

**Fig. 6:**
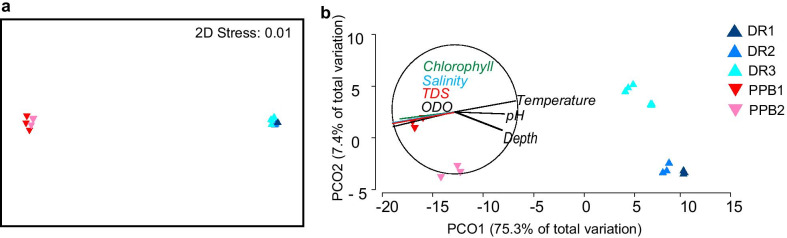
Diversity of the Davies Reef (DR) and Port Phillip Bay (PPB) sample assemblages. Explored by **a** nMDS and **b** PCO based on Bray–Curtis dissimilarity matrices. Environmental variables displayed in vector overlay are dissolved oxygen (ODO), salinity, total dissolved solids (TDS), and chlorophyll a on left side of vector, and temperature, pH and depth on right side of vector.

Non-metric multidimensional scaling (nMDS) results indicated clear separation between samples based on location, with all samples at each location tightly grouped (Fig. 6a). Principle coordinate analysis (PCO) indicated a strong correlation between the overall assemblage structures and the measured environmental variables optical dissolved oxygen (ODO), salinity, TDS, chlorophyll, pH, and temperature), with replicates for each sample site grouped and clear separation between samples based on location (Fig. 6b). The analysis of similarity (ANOSIM) comparing sequence abundances between locations resulted in *R* = 1, *P* = 0.001, indicating ‘perfect’ separation of sequence abundance based on location. It was determined by similarity percentages species contributions (SIMPER) that the average dissimilarity between the assemblages at the two locations was 24.01% (Table 2). Over 140 species contributed to the variation, with the top 5 contributors including *Warnowia sp*., *Paragymodinium shiwahaense*, and *Gyrodinium helveticum*, summarised in Table 2.

**Table 2:** Dissimilarity within and between the two locations determined by SIMPER for the whole assemblage, and subsets bioluminescent and HAB species. The top 5 contributors to between-site variation are listed, with the percentage contributed in parentheses

	Whole assemblages		Bioluminescent subset		HAB subset	
Average dissimilarity between sites (%)	24.01		22.88		24.24	
Number contributors	147		18		30	
Top 5 contributor(s) (%)	*Warnowia sp.*	(1.61)	*Lingulodinium polyedra*	(8.58)	*Karlodinium veneficum*	(4.92)
	*Paragymodinium shiwahaense*	(1.27)	*Protoperindinium pellucidum*	(7.33)	*Gymodinium sp.*	(3.49)
	*Nematodinium sp.*	(1.09)	*Pyrocytis lunula*	(4.81)	*Phalacroma capa*	(3.34)
	*Protoerythropsis sp.*	(1.00)	*Polykrikos schwartzii*	(4.75)	*Phalacroma mitra*	(3.33)
	*Gyrodinium helveticum*	(0.96)	*Alexandrium monilatum*	(4.66)	*Prorocentrum rhathymum*	(3.24)
Average dissimilarity of replicates within sites (%)						
Davies Reef		8.46		12.35		8.14
Port Phillip Bay		11.00		17.38		8.99

**Fig. 7:**
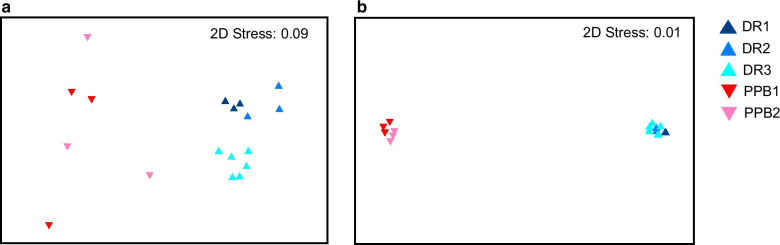
Diversity of the Davies Reef (DR) and Port Phillip Bay (PPB) subset assemblages. Explored by nMDS based on Bray–Curtis dissimilarity matrices. **a** Bioluminescent subset nMDS. **b** HAB subset nMDS

nMDS results indicated that although there was separation for both assemblage subsets based on location, there was higher within-site variation for the bioluminescent subset compared to the HAB subset (Fig. 7). Nonetheless, both subsets differed significantly between locations, with ANOSIM *R* = 0.945 (*P* = 0.001) and R = 1 (*P* = 0.001) for the bioluminescent and HAB subsets, respectively. SIMPER results further supported higher within-site variability for the bioluminescent subset, with the average dissimilarity within DR being 12.35% for bioluminescent compared to 8.14% for HAB, and within PPB being 17.38% for bioluminescent compared to 8.99% for HAB (Table 2). SIMPER results also showed that the average dissimilarity between locations was 22.88% for the bioluminescent subset and 25.99% for the HAB subset, with 18 and 29 species contributing to the dissimilarity between locations, respectively. The top 5 contributors to the dissimilarity for each subset are summarised in Table 2.

Distance-based linear models (DistLM) determined that 88% of the total variation in the entire assemblages at the two locations was best explained by three environmental variables: salinity, temperature, and ODO (Table 3). In contrast, for the bioluminescent subset, only the two variables salinity and ODO significantly explained the assemblage differences (accounting for 56% of the variation); whereas, for the HAB subset, the four variables salinity, temperature, ODO and depth were significant and explained 92% of the variation (Table 3). Importantly, 1) salinity was the strongest explanatory factor for both subsets of assemblages, and 2) temperature and depth only explained HAB assemblages, not bioluminescent assemblages.

**Table 3:** Distance-based linear models (DistLM) for determination of environmental variables that explain assemblage variation. Variables included in the sequential analysis were Chlorophyll a, ODO, salinity, TDS, pH, temperature and depth, with only terms that remained in the best fit model presented

Variable	Pseudo-F	*P*-value	% variation
Whole assemblage (R^2^ = 0.88; AICc = 63.47)			
Salinity	42.97	0.001	72.87
Temperature	7.30	0.001	8.88
ODO	7.02	0.001	6.09
Bioluminescent subset (R^2^ = 0.56; AICc = 84.77)			
Salinity	12.84	0.001	44.51
ODO	4.09	0.001	11.89
HAB subset (R^2^ = 0.91; AICc = 60.59)			
Salinity	55.92	0.001	77.75
Temperature	6.06	0.001	6.40
ODO	6.24	0.002	4.89
Depth	4.02	0.002	2.59

*ODO* optical dissolved oxygen, *TDS* total dissolved solids

## Discussion

Our aim was to assess the diversity and composition of dinoflagellate assemblages at two different Australian locations using both conventional microscopy and eDNA sampling with HTS genotyping. It was expected that both methods would detect similar genera, with HTS potentially detecting broader diversity than conventional methods. Although widely reported for other regions such as Europe and North America, the use of HTS for assessing diversity and composition of dinoflagellates in Australian waters is less common. HTS is typically utilised for detection of one species rather than for an overall assessment of dinoflagellates assemblages. We have demonstrated the feasibility of utilising eDNA sampling with HTS genotyping for assessment of dinoflagellate diversity across different locations.

Microscopy-based taxonomic analysis of environmental samples found that the two locations had low numbers of dinoflagellates compared to diatoms and other flagellates (Additional file 1: Tables S1 and S2). The dinoflagellate diversity was restricted to 17 genera. HTS confirmed the presence of these genera but identified much greater diversity—around 150 genera in total. However, the results here for the two most abundant dinoflagellate genera (*Gyrodinium* and *Gymnodinium*) concurred with the molecular results. Traditional methods using light microscopy can be prone to underestimating the diversity of dinoflagellate species; particularly for athecate (i.e. lacking thecal plates or unarmoured) dinoflagellates, identification is often only possible to the order or genus level due to fragile structural components being damaged during collection (e.g. netting) and fixation [42]. Due to the level of underestimation, inferences derived from traditional methods may lead to mischaracterisation of the evolutionary history and ecology of the organisms at these marine locations. Comparisons between traditional surveys of marine organisms and more recent molecular surveys utilising eDNA coupled with HTS have shown that although the identified taxa may vary, the differences arise due to advantages and disadvantages of each method and, overall, the methods are complementary [43, 44].

The eukaryotic markers used here revealed a high level of diversity within the total assemblages at both locations. The application of HTS showed the ability to detect even trace levels of dinoflagellates amongst the other taxa contained in these planktonic communities [15]. The use of HTS has shown that protists, and dinoflagellates in particular, have high levels of diversity within marine communities [21, 27].

Tropical waters of DR were represented by 11 superclades from which Gymnodiniales sensu stricto*,* and Gyrodinium showed 64% relative abundance of sequence reads. In contrast, the cold waters of PPB were dominated a single genus, *Gyrodinium*, with abundances of 82% of sequence reads. Typically prevalent in coastal waters, *Gyrodinium* species are globally ubiquitous, unarmoured (athecate) heterotrophs [1, 45]. Like all dinoflagellates, *Gyrodinium* spp. exhibit slow growth rates compared to other phytoplankton (e.g. ciliates, diatoms) [46].

A very low percentage of the dinoflagellate assemblage was identified as being bioluminescent species, especially considering sampling was undertaken either during or following bioluminescent events (Table 1, Fig. 4). However, the results here indicate that *N. scintillans* was present in very low numbers at both sites, with the most abundant bioluminescent species being *Polykrikos kofoidii*, a heterotrophic, unarmoured, free-living bioluminescent dinoflagellate [47] (Table 1). There could have been other sources of bioluminescence in the water from other organisms such as bacteria that were not sequenced by the 18S rRNA  primers used.

The list of bioluminescent and HAB-related species was developed by comparison of the DinoREF database to the wider literature and is by no means exhaustive. It must be noted that these two ‘subsets’ are not mutually exclusive, with eleven species belonging to both HAB and bioluminescent subsets (Table S6). Further, several 18S rRNA sequences of bioluminescent dinoflagellate species detected in previous studies of South-Eastern Australian waters are missing from the DinoREF database. These included two species for which we added reference sequences (*Noctiluca scintillans* and *Pyrocytis noctiluca*) and one for which there was not a sequence available (*Pyrocystis fusiformis)*. Thus, in order to assess the bioluminescent species present in dinoflagellate assemblages in Australian waters, further development of the DinoREF database by the addition of more reference species is required. For better identification of bioluminescent species, 18S rRNA-based data could be complemented by genotyping based on variations in *lcf*. Both general and species-specific *lcf* gene primers are available [19, 48–51]. However, non-bioluminescent strains of *Alexandrium tamarense*, *Ceratocorys horrida* and *Noctiluca scintillans* were demonstrated to contain the *lcf* gene [8, 50], indicating that although useful, further methods to confirm bioluminescence are needed. Transcriptomics alongside proteomics revealed that *P. lunula* contains the gene for, and actively expresses, luciferase binding protein, not previously observed for this species [52]. Extending the use of ‘omics’ technologies to other bioluminescent dinoflagellates will help reveal the relationships between bioluminescent taxa and the expression and/or activity of bioluminescence.

HAB-related dinoflagellates can be divided into five groups: 1) high-biomass blooming, non-toxic species that directly or indirectly kill marine species via deoxygenation of water or other physical effects upon the water column; 2) food-poisoning toxin producers causing either neurological or gastrointestinal symptoms in humans; 3) those not harmful to humans, but harmful to marine organisms via mechanical effects such as gill damage, or haemolytic compound secretion; 4) toxin producers harmful to humans, particularly in aerosols; and 5) those listed as not toxic to humans but shown to produce toxins, thus potentially dangerous [1, 4, 53–55]. Using these definitions, almost half the total assemblage from PPB was identified as HAB-related, being dominated by *Gyrodinium spirale* and *G. dominans* (Fig. 5). These two species were also dominant within the HAB subset at DR, although not to the same extent as at PPB. Due to the potential economic and health impacts of HABs, DinoREF was screened for sequences of potentially toxic dinoflagellates [2, 27, 32, 56]. PPB is subject to high levels of direct and indirect anthropogenic influences, which could explain the up-to 1.6 fold higher representation of identified HAB-related taxa.

The representation of HAB-related genera did not correspond to their respective species diversity. The most abundant genus, *Gyrodinium* (16–42%), was represented just by 2 species from the possible 7 described in the DinoREF database. In contrast, one of the least abundant genera, *Alexandrium* (0.05–0.1%), was comprised of 17 species from the possible 23 contained in the DinoREF database, however all 23 *Alexandrium* species were identified in the whole assemblages (Additional file 1: Table S4 and S5). Of the species identified here, 11 are previously reported from Australian waters [16, 57, 58]. There are also many reports of *Alexandrium catenella*, Group IV, in Australian waters, a known HAB associated with paralytic shellfish poisoning; however, this species is not contained in the DinoREF database. Still, there were 13 *Alexandrium* species detected that were not previously known to be present in Australian marine waters, including *A. andersonii, A. cohorticula, A. hiranoi, A. insuetum, A. leei, A. mediterraneum,* Group II, *A. monilatum, A. pohangense, A. satoanum, A. tamarense* Group III, *A. tamiyavanichii, A. tamutum* and *A. taylorii.* Another HAB species not previously reported in Australian waters but detected at low relative abundances here is *Karenia brevis* [58]. These novel findings clearly highlight the usefulness of the ability to be able to detect rare and less abundant taxa utilising eDNA sampling and HTS genotyping.

The significant variation of assemblage structures between locations (Figs. 6, 7; Table 2) and their strong correlations with environmental variables (Table 3) suggest that species presence and relative abundance of sequence reads are constrained by temperature and salinity [59]. Interestingly, while the dinoflagellate assemblage as a whole was explained to 88% by the measured environmental parameters, the explanatory power diverged when separating out the bioluminescent and HAB-related subsets: HAB assemblages were explained to 92%, whereas bioluminescent assemblages were explained only to 56%. This result suggests that the distribution and relative abundances of HAB species are more strongly influenced by environmental factors than are bioluminescent species. However, the distribution and abundance of bioluminescent species maybe affected by other parameters that were not measured such as the presence of other prey and/or predator species. The result is also consistent with the higher within-site variability observed for the bioluminescent assemblage compared to the HAB assemblage regardless of location (Fig. 7; Table 2). However, the ecological mechanisms producing this pattern are unknown, and elucidation would require further temporal surveys. The stronger environmental influence on HAB assemblages is also reflected by their correlation with four parameters (salinity, temperature, ODO, and depth), whereas bioluminescent assemblages correlated with only two (Table 3). The first three parameters explaining HAB assemblages (salinity, temperature and ODO) are all influenced by anthropogenic activity, consistent with studies indicating that HABs are triggered by human impacts (reviewed by [2]). It is important to note that nutrients considered important stressors for dinoflagellate growth, such as nitrogen and phosphate, were not measured here. Regardless, our finding that HAB species were more predominant at PPB than at DR is also consistent with the extensive anthropogenic impacts documented in PPB [41]. In contrast, neither temperature nor depth were significant explanatory factors for bioluminescent assemblages, and indeed the ecological and environmental constraints on the distribution of bioluminescent species are poorly known in comparison, although recent studies have begun to elucidate some of these relationships, for example, *Noctiluca scintillans* tolerates a wide range of salinity and temperatures [35].

The species that differed most between locations (top five including *Warnowia sp*., *Paragymodinium shiwahaense*, and *Gyrodinium helveticum* shown in Table 2) are the ones most likely to be strongly impacted by environmental variation since those that contributed less to dissimilarity are essentially distributed more evenly. However, the differences in distribution may reflect differences more broadly in biogeographic ranges that are related to other factors not measured here. Furthermore, the data here are from a single time point, whereas any phenological differences in population dynamics among species could cause assemblage differences to fluctuate seasonally. As PPB is a temperate latitude embayment, it experiences stronger spring plankton blooms than DR within the tropics, thus the samples collected during Austral winter likely represent a seasonal low point at PPB. A temporal study of assemblage structure with HTS genotyping and measurements of environmental factors expanded to include nutrients such as nitrogen and phosphate could provide more insight into the constraints on both HAB and bioluminescent assemblages.

## Conclusions

- Application of HTS in genotyping enables the detection of rare and less abundant taxa compared to traditional methods. Further, all taxa identified using microscopic techniques were detected using HTS genotyping. However, HTS genotyping detected approximately ninefold higher numbers of taxa compared to traditional methods and expanded upon the previously described diversity for these two locations.
- Comprehensive microscopy-based and molecular taxonomic assessments of two different Australian ecosystems revealed a greater diversity of dinoflagellates in the warm waters of DR (with eleven superclades, from which Gymnodiniales sensu stricto and Gyrodinium comprised 33% and 34% of sequence reads, respectively), compared to the cooler waters of PPB that were dominated by the superclade Gyrodinium (82%).
- Up to 1.5-fold higher concentrations of HAB-related species were identified in PPB compared to DR, possibly reflecting the greater anthropogenic influences in this area.
- Bioluminescent species were represented by only up to 0.23% of total sequence reads.

## Methods

### Sample collection

DR (Great Barrier Reef, Queensland; GPS: -18.8284 147.6354) samples were collected over a 4-day period: day 1 (three replicate samples from site DR1), day 2 (three replicate samples from site DR2), and day 4 (six replicate samples from site DR3). PPB samples were collected on a single night from two sites (three replicate samples from each site, PPB1 and PPB2) (GPS: -38.2759 144.8304 for station PPB1 and -38.3274 144.9034 for station PPB2). Water samples were collected using a 15 L Niskin bottle at discrete depths within the water column, selected to be within the range of observed bioluminescence. Depths of sampling at DR were 15 m (DR1) and 10 m (DR2 & 3), while all samples at PPB were taken at 8 m. For microscopical identification of species, a 1 L collection bottle was filled and preserved using a final concentration of 0.1–0.5% Lugol’s iodine solution and stored in a dark, cooled insulated container. Microscopical identification was performed by Microalgal Services, Ormond, Victoria, Australia [60]. For molecular identification of species, samples were prefiltered through a 50 µm mesh sieve to remove large organisms and/or debris, then 6–8 L were filtered through Whatman glass fiber filters (0.45 µm pore size, 47 mm diameter) under vacuum. All filters were placed in 15 mL Falcon tubes and stored at − 20 °C, either immediately or transferred to the laboratory on ice in a dark insulated container then frozen, until processed. To obtain a profile of the water column, an EXO1 Multiparameter Sonde (YSI) was used with measurements recorded typically for both down and upcast. Parameters measured included chlorophyll a, conductivity, depth, ODO, redox potential, salinity, phycoerythrin fluorescence (indicating cyanobacteria), TDS, pH, temperature and GPS latitude and longitude.

### DNA extraction and sequencing

DNA was isolated from filters using an optimised CTAB method [61]. Filters were immersed in 500 µL of pre-warmed (65 °C) CTAB isolation buffer (2% CTAB (Sigma, Saint Louis, USA), 1.4 M NaCl, 100 mM Tris pH 8.0, 20 mM EDTA PVP, 0.01% w/v SDS, 0.2% mercaptoethanol). Samples were vortexed for 5 min, then briefly cooled on ice before the addition of a 1:1 solution of chloroform and isoamyl alcohol (24:1 v/v) (Sigma, Saint Louis, USA). Samples were centrifuged at ≈ 12,000 x*g* for 10 min and the supernatant collected and re-extracted twice as described. Samples were cooled on ice for 5 min, then centrifuged at ≈ 12,000 x*g* for 15 min. DNA pellets were washed with 500 µL of 70% ethanol, centrifuged at ≈ 12,000 x*g* for 6 min, and air-dried. DNA was re-suspended in 50 µL of DNase-free water and stored at 4 °C overnight for the complete dissolution of DNA. The quantity and purity of template DNA was assessed using a Nanodrop spectrophotometer (PicoDrop Ltd, Hinxton, UK). The dinoflagellate 18S rRNA gene was amplified utilising primers based on Zhang, Bhattacharya [62]: 18ScomF1 (5′-GCTTGTCTCAAAGATTAAGCCATGC-3′) and Dino18SR1 (5′-GAGCCAGATRCDCACCCA-3′) using 1 µL of extracted DNA (100 ng/µL). PCR was undertaken with an initial denaturation at 95 °C for 3 min, followed by 40 cycles of denaturation at 95 °C for 35 s, annealing at 53 °C for 40 s and extension at 72 °C for 1 min with final extension at 72 °C for 5 min. 18S rDNA amplicons (≈ 1.75 kb) were sequenced by the Australian Genome Research Facility (www.https://www.agrf.org.au/) using paired-end Illumina sequencing.

### Data analyses

A dinoflagellate (Dinophyceae) 18S rRNA reference database was created by downloading DinoREF (Pub med REF PMID: 29603631), containing a non-redundant curated set of 422 species, and adding to it the 18S rRNA sequences of *Noctiluca scintillans* (GenBank: KR527331.1) and *Pyrocystis noctiluca* (GenBank: AF022156.1) [63], which were of interest and absent from DinoREF. Raw fastq files were pre-processed using BBDuk [64], removing adaptor and poor quality sequences. The following parameters were used with BBDuk: “ktrim = r k = 23 mink = 11 hdist = 1 tpe tbo ftl = 31 trimq = 15”. This included a hard trim of 31 bases to remove adaptor, kmer-trimming, primer (dimer) trimming, as well as quality trimming (to Q15). Pre-processed fastq files were aligned against the 18S rRNA reference using Burrows-Wheeler Alignment (bwa mem v0.7.17) tool [65]. Conversion into BAM format and extraction of uniquely mapped reads was carried out using SAMtools [66]. Chimeric and multimapped reads were filtered out via SAMtools with using the “SA” tag and -bq1 command. SAMtools was further used to count reads aligned to each dinoflagellate species.

The DinoREF analysis resulted in an Excel spreadsheet containing the number of sequence reads that aligned to each entry, as well as a taxa file. Excel was used to determine the relative abundance of sequence reads as a percentage, using formula 1, and generate profiles of the total dinoflagellate assemblage for each location (i.e. average of DR and average of PPB).

Formula 1: $$\left(\frac{no\;of\;sequnce\;reads}{total\;sample\;sequence\;readss}\right)\times100\%$$  

For environmental data, YSI data were exported to Excel. The relevant environmental data were selected by using the average measurements over a 1 m range corresponding to the depth of the plankton sample. For example, for plankton samples taken at 10 m, environmental measurements from 9.6 to 10.5 m were selected and averaged.

Relationships between biological and environmental data were tested with PRIMER and PERMANOVA + . The numbers of sequences were treated as abundances. Status of species as bioluminescent or HAB-related were added as indicators. Environmental variables were chlorophyll a (µg/L), ODO (mg/L), salinity (psu), TDS (mg/L), pH, temperature (°C) and depth (m). Environmental data were normalised, and a resemblance matrix based on Euclidean distance was generated. In order to capture both highly represented and rarely represented species, biological data were transformed to the fourth root, and a resemblance matrix based on Bray Curtis similarity was generated. Relationships between biological and environmental data were analysed by nMDS and PCO, with the significance of separation determined by ANOSIM. SIMPER was used to determine the taxa that contributed most to the variation. To determine the environmental variables that explained the relationships, a DistLM with stepwise selection criteria of Akaike Information Criterion (AICc) regression was used, allowing for identification of predictor variables that contributed significantly to the assemblage variation between the two locations. Analyses of the bioluminescent and HAB subsets were undertaken as for the whole assemblages. A taxa file for species, genus, family, and order was included for analyses of taxa within the assemblages. A second taxa file for species and superclade, as determined by Mordret et al. [32], was also included. Biological data were analysed at different taxonomical levels using AGGREGATE function and shade plots.

## Supplementary Information

**Additional file 1: Table S1.** Taxonomic identification of the main planktonic representatives, diatoms and dinoflagellates at Davies Reef. X denotes species observed in sample during initial microscopic examination but not encountered during cell counting. **Table S2**. Taxonomic identification of the main planktonic representatives, diatoms and dinoflagellates at Port Phillip Bay. X denotes species observed in sample during initial microscopic examination but not encountered during cell counting. **Table S3**. Taxonomy and relative abundance of sequence reads (%) for all sample replicates. DR: Davies Reef; PPB: Port Phillip Bay. **Table S4.** Mean relative abundance of sequence reads of dinoflagellate superclades, genera and species as defined by Mordret et al. (2018) at Davies Reef (DR) and Port Phillip Bay (PPB). The superclade Noctilucaphyceae was added to reflect the addition of *Noctiluca scintillans* to the DinoREF database. **Table S5.** Mean relative abundance of sequence reads (%) of dinoflagellate species identified at Davies Reef (DR) and Port Phillip Bay (PPB). **Table S6.** List of bioluminescent and HAB species identified within the two dinoflagellate assemblages. HAB species are assigned numbers 1-5, based on the typologies as defined by Lassus et al. [1, 4, 53–55]. **Table S7.** Mean relative abundance of sequence reads (%) of HAB-related species at Davies Reef (DR) and Port Phillip Bay (PPB).

## Data Availability

All data generated or analysed during this study are included in this published article (and its supplementary information files).

## Abbreviations

ANOSIM: Analysis of similarity
COI: Cytochrome c oxidase 1
DistLM: Distance based linear models
DR: Davies Reef
HAB: Harmful algal blooms
HTS: High throughput sequencing
ITS2: Nuclear ribosomal internal transcribed spacer region
nMDS: Non-metric multi-dimensional scaling
ODO: Optical dissolved oxygen
PCO: Principle coordinate analysis
PPB: Port Phillip Bay
SIMPER: Similarity percentages-species contributions
TDS: Total dissolved solids
